# Digital clock assessment in South Asian setting: pilot study

**DOI:** 10.1038/s44400-025-00056-6

**Published:** 2026-02-02

**Authors:** Ram Jagannathan, Deepa Mohan, Rani Komal, Dimple Kondal, Poongothai Subramani, Priyanka Menon, Rima Pai, Ranjith Mohan Anjana, Mohammed K. Ali, Sailesh Mohan, Suvarna Alladi, Nikhil Tandon, Dorairaj Prabhakaran, Viswanathan Mohan, Allan I. Levey, K. M. Venkat Narayan, Felicia C. Goldstein

**Affiliations:** 1 Emory Global Diabetes Research Center, Woodruff Health Sciences Center and Emory University, Atlanta, GA, USA.; 2 Hubert Department of Global Health, Emory University, Atlanta, GA, USA.; 3 Madras Diabetes Research Foundation (ICMR Collaborating Centre of Excellence) and Dr Mohan’s Diabetes Specialities Centre, IDF Centre of Excellence in Diabetes Care, Chennai, India.; 4 Centre for Chronic Disease Control, New Delhi, India.; 5 University of Augusta, Augusta, GA, USA.; 6 Department of Family and Preventive Medicine, Emory University School of Medicine, Atlanta, GA, USA.; 7 National Institute of Mental Health and Neurosciences, Bangalore, India.; 8 All India Institute of Medical Sciences, New Delhi, India.; 9 Department of Neurology, Emory University School of Medicine, Atlanta, GA, USA.; 10 Emory Goizueta Alzheimer’s Disease Research Center, Atlanta, GA, USA.

## Abstract

As cognitive impairment increasingly burdens low- and middle-income countries, scalable tools for early detection are pivotal. This pilot study evaluated the feasibility and performance of a tablet-based digital Clock Drawing Test (DCTclock) in 303 adults aged ≥ 50 years from the population-based CARRS cohort in urban India. Participants completed both the tablet-based DCTclock and the paper-based Mini-Cog, which was used to classify cognitive status (impaired: ≤2 vs. unimpaired: ≥3). The DCTclock required under four minutes to administer, and 99.3% of tests yielded analyzable data. Compared with cognitively unimpaired participants (n = 252), those classified as impaired (n = 51) scored significantly lower on the DCTclock total score and subdomains, particularly spatial reasoning and information processing. Performance was lower with older age and lower educational attainment (both p < 0.001) but did not differ by sex. The DCTclock demonstrated moderate discriminative accuracy for Mini-Cog-defined impairment (AUC = 0.669), and each interquartile range higher total score was associated with 52% lower odds of impairment (OR = 0.48; 95% CI, 0.32–0.70). Similar patterns were observed using FDA-recommended thresholds, while in this cohort, a ROC-derived cut point of 38 yielded slightly stronger discrimination (3.95-fold greater odds). These findings support the DCTclock’s feasibility and potential as a scalable digital tool for community-based cognitive screening.

Alzheimer’s disease and related dementias (AD/ADRD) currently affect an estimated 57.4 million people globally, a figure projected to exceed 152 million by 2050^1^. Approximately 70% of this future burden is expected to occur in low- and middle-income countries, with South Asia experiencing one of the steepest increases due to demographic aging and shifting epidemiological patterns^2^. Since AD pathology develops years or even decades before the onset of clinical symptoms, midlife represents a critical window for identifying individuals at elevated risk and initiating early intervention^3^.

There is a pressing need for scalable, psychometrically robust tools that can detect early cognitive changes across diverse populations. The Clock Drawing Test (CDT) is widely used in clinical and research settings as a brief screening instrument that engages multiple cognitive domains, including executive function, visuospatial organization, and graphomotor control^4^. However, its broader utility is hindered by methodological variability. The absence of standardized administration and scoring protocols has led to the proliferation of heterogeneous systems that differ in complexity and interpretation thresholds, limiting reproducibility and comparability across studies^5^. Besides, many scoring approaches rely on subjective criteria, which reduces inter-rater reliability and diagnostic precision, particularly in settings without specialized expertise. Tools, such as the Mini-Cog^6^, which incorporates the CDT alongside a word recall task, face similar limitations, including inconsistent administration and reduced applicability in linguistically and educationally diverse populations^7,8^.

Digital adaptations of the CDT, such as the DCTclock (Linus Health), have been developed to address these limitations^9,10^. These tools capture high-resolution temporal and spatial data, enabling detailed analysis of cognitive performance. Features, such as stroke sequencing, drawing latency, and the amount of time-in-air provide quantitative markers of early impairment that traditional paper-and-pencil methods fail to capture. With automated scoring and standardized administration, digital CDT platforms enhance measurement precision and reduce dependence on rater expertise and clinical judgment, supporting broader implementation across varied healthcare environments.

Extensive validation studies in U.S.-based cohorts have demonstrated the clinical utility of DCTclock in detecting early cognitive decline^9^. The overall DCTScore and the test-derived features have been associated with APOE genotype or the AD polygenic risk score^11^, as well as plasma AD biomarkers, amyloid and tau PET burden^12–14^, and MRI-derived brain volumetrics^15^. The DCTclock shows strong discrimination between normal cognition and mild cognitive impairment (AUC = 0.86), although this is lower than the Preclinical Alzheimer Cognitive Composite (AUC of 0.95). However, among cognitively normal older adults, DCTclock performance is more strongly associated with amyloid burden (Cohen’s d = 0.76 vs. 0.30 for PACC), suggesting greater sensitivity to preclinical Alzheimer-related changes^14^.

However, the feasibility and acceptability of DCTClock in low- and middle-income countries remain largely uncharacterized. This gap is particularly salient in South Asia, where linguistic heterogeneity, educational disparities, and varying levels of digital literacy may present unique challenges for implementation^16^. This pilot study aimed to evaluate the feasibility and performance of DCTclock in a demographically diverse South Asian cohort. We also examined the extent to which DCTclock scores vary by age, sex, and education, and assessed their ability to discriminate Mini-Cog-defined cognitive impairment. We further analyzed domain-specific DCTclock metrics under both command and copy conditions to determine which aspects of cognition are most sensitive to early deficits in this population. Findings provide preliminary evidence on the implementation potential and discriminatory utility of tablet-based cognitive screening in low-resource, heterogeneous settings.

## Results

Participant characteristics of the study sample by Mini-Cog status are shown in Table 1. A total of 303 participants aged 50 years or older were included (mean age 62.3 ± 8.9 years; 55.4% women). Based on Mini-Cog screening, 51 participants (16.8%) were classified as cognitively impaired and 252 (83.2%) as unimpaired.

Participants with cognitive impairment were older on average, and a larger proportion were aged above 70 years. Women comprised a greater proportion of the impaired group compared to the unimpaired group. Educational attainment and city of residence were broadly similar between groups. Only two participants, both in the impaired group, reported current memory concerns or use of memory-related medications.

DCTclock was completed in approximately 3–4 min per participant. Nearly all assessments yielded analyzable data, with only 0.7% excluded due to incomplete or unscorable drawings. In contrast, Mini-Cog administration required approximately 6 min on average. While nearly all participants completed the total score component, 47 individuals (15.5%) declined or were unable to complete the clock drawing.

**Fig. 1 |:**
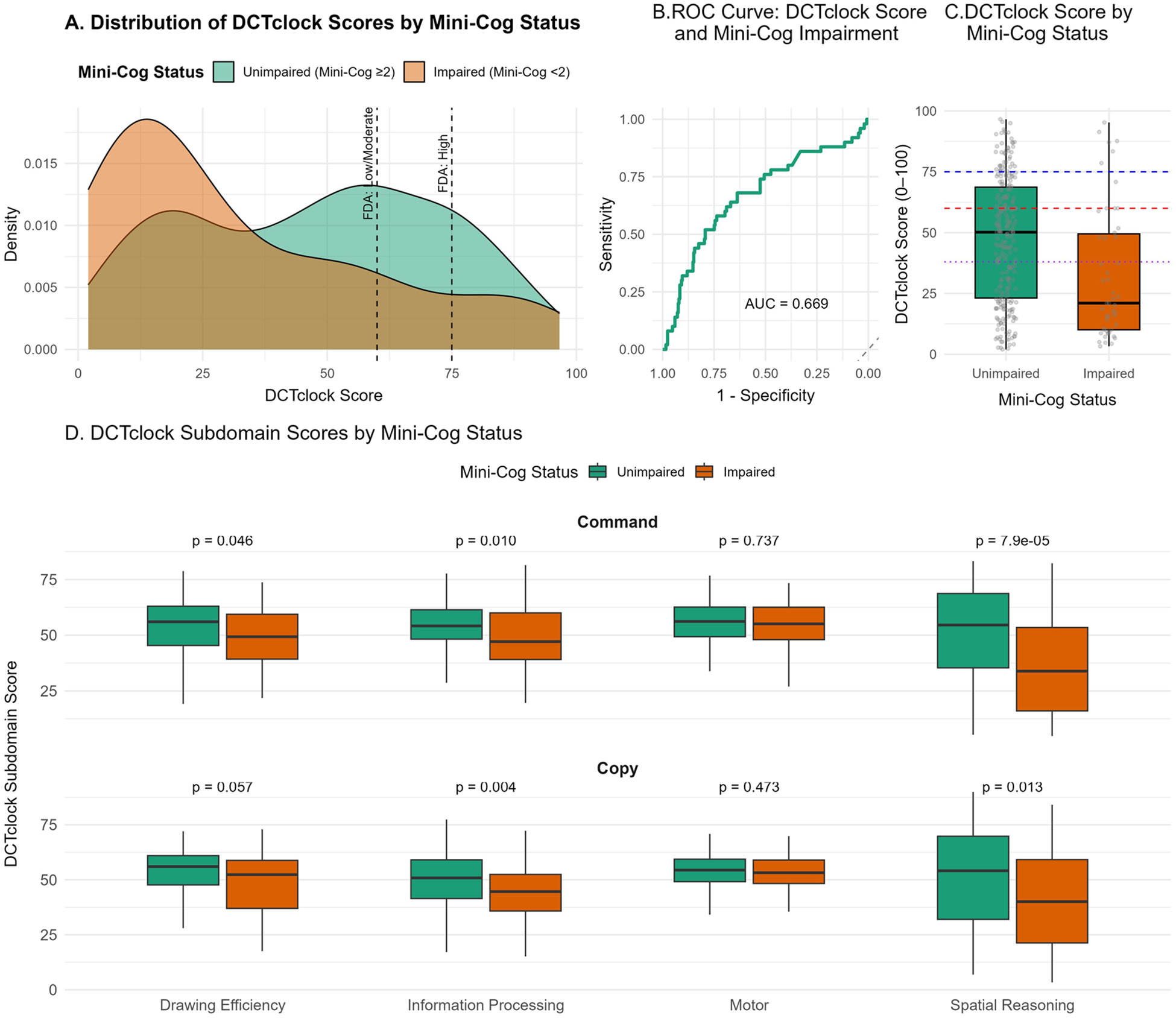
DCTclock Scores and Subdomains in Relation to Mini-Cog Status. **A** Distribution of total DCTclock scores by Mini-Cog status. Dashed lines at scores of 60 and 75 indicate thresholds used in prior validation studies. **B** Receiver operating characteristic (ROC) curve showing the discriminatory ability of the DCTclock score to identify Mini-Cog impairment (AUC = 0.669 (95%CI: 0.580–0.758)). **C** Boxplot comparing total DCTclock scores between Mini-Cog impaired and unimpaired participants. Dashed lines represent key thresholds: clinical cutoffs at 60 (red) and 75 (blue), and ROC-derived optimal threshold at 38 (purple dotted). **D** Comparison of DCTclock subdomain scores (Command and Copy conditions) by Mini-Cog status. Domains include Drawing Efficiency, Information Processing, Motor, and Spatial Reasoning. P-values are from Wilcoxon rank-sum tests comparing impaired vs. unimpaired groups. **Mini-Cog Status:** □ **Unimpaired** = Mini-Cog total score ≥3 □ **Impaired** = Mini-Cog total score ≤2.

Total DCTclock scores were substantially lower among participants with Mini-Cog-defined impairment. The score distribution showed clear separation between groups, with impaired participants clustering below conventional cutoffs of 60 and 75 (Fig. 1A–C). Subdomain scores were also consistently lower among the impaired group, particularly for information processing and spatial reasoning under both command and copy conditions (Fig. 1D).

### DCTclock Performance by Demographic Characteristics

Associations between age, sex, and educational attainment with DCTclock performance are shown in Table 2. Total DCTclock scores were progressively lower with higher age, with participants aged 61–70 and over 70 scoring 10.9 and 21.3 points lower, respectively, than those aged 50–60. Age-related differences were observed across both command and copy conditions and were most pronounced for spatial reasoning, particularly in the copy domain, where scores were more than 16 points lower in the oldest group.

Educational attainment was strongly associated with performance across nearly all domains. Participants with college or graduate-level education scored significantly higher than those with a high school education or less, with the largest differences observed in information processing and spatial reasoning. Drawing efficiency also varied by education level, whereas motor subdomain scores showed little variation.

Sex-based differences were modest. Female participants scored slightly lower than males in information processing and drawing efficiency within the copy condition; however, the differences were small in magnitude and inconsistent across other domains.

### Discriminative Performance of DCTclock

The discriminative performance of the DCTclock score for Mini-Cog-defined cognitive impairment was assessed using ROC analysis (Fig. 1B). The AUC was 0.669 (95% CI: 0.580–0.758), indicating moderate classification accuracy. The optimal threshold based on Youden’s index was 37.4, yielding a sensitivity of 68.0% and a specificity of 63.7%. Although widely used clinical thresholds of 60 and 75 captured general trends, the ROC-derived cut-point more accurately distinguished impaired from unimpaired individuals. Subdomain analyses similarly demonstrated significantly lower scores among impaired participants across key cognitive dimensions, highlighting the multidomain sensitivity of the DCTclock assessment.

### Association of DCTclock Metrics with Mini-Cog Impairment

**Fig. 2 |:**
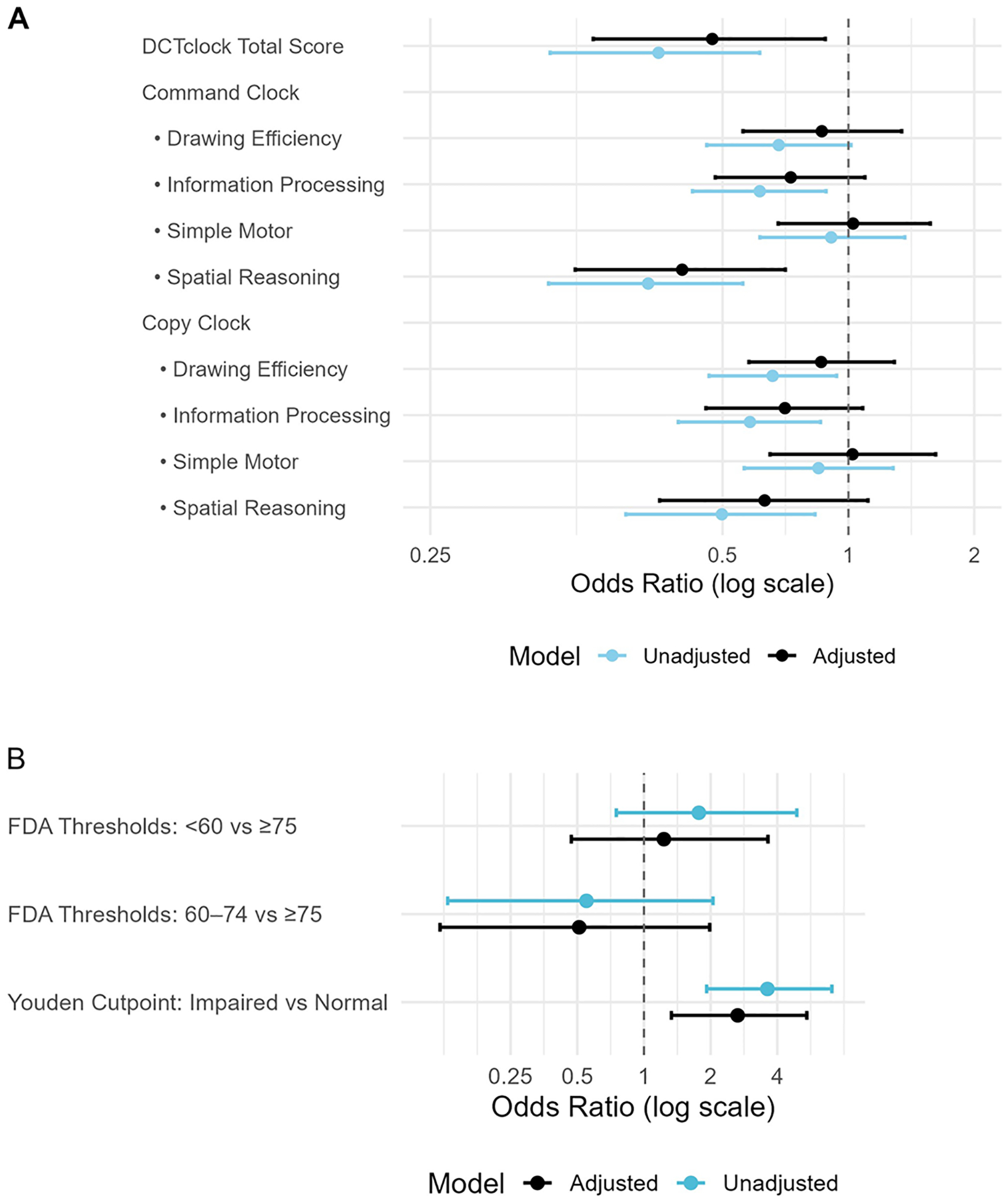
Associations Between DCTclock Performance and Mini-Cog Cognitive Impairment Status. **A** Odds ratios for cognitive impairment by DCTclock metrics. **B**. Odds Ratios for Cognitive Impairment DCTclock total score categories. (**A**) Forest plot showing unadjusted and adjusted odds ratios (ORs) with 95% confidence intervals (CIs) for Mini-Cog impairment (Mini-Cog score ≤2 vs. ≥3) by overall and subdomain DCTclock metrics. The DCTclock total score and subdomains (Drawing Efficiency, Information Processing, Simple Motor, and Spatial Reasoning) were analyzed separately under Command and Copy conditions. All continuous exposures were scaled by their interquartile range (IQR). Blue lines represent unadjusted models and black lines represent models adjusted for age category, sex, and education. ORs below 1.0 indicate that better cognitive performance is associated with lower odds of impairment. **B** Forest plot displaying associations between Mini-Cog impairment and categorical DCTclock scores based on (i) U.S. FDA-cleared thresholds (<60, 60–74, ≥75) and (ii) a data-derived cutpoint using Youden’s index from the ROC curve. The ≥75 group (FDA) and “Normal” group (Youden) were used as reference categories. Blue lines represent unadjusted models and black lines represent models adjusted for age category, sex, and education.

In models adjusted for age, sex, and educational attainment (Fig. 2A), higher DCTclock total scores were associated with substantially lower odds of Mini-Cog-defined cognitive impairment. Each interquartile range (IQR) higher total score corresponded to an adjusted OR of 0.48 (95% CI, 0.32–0.70) of Mini-Cog-defined cognitive impairment. Similar inverse associations were observed across key subdomains in the command clock domain, with adjusted ORs of 0.53 (95% CI, 0.35–0.79) for drawing efficiency, 0.57 (95% CI, 0.39–0.84) for information processing, and 0.52 (95% CI, 0.36–0.76) for spatial reasoning. In contrast, simple motor performance under command conditions was not associated with cognitive status.

Subdomain scores from the copy condition yielded comparable findings. Adjusted ORs were 0.55 (95% CI, 0.37–0.83) for drawing efficiency, 0.58 (95% CI, 0.40–0.85) for information processing, and 0.49 (95% CI, 0.33–0.72) for spatial reasoning. As in the command condition, copy-based motor scores were not significantly associated with impairment (OR, 0.85; 95% CI, 0.58–1.26).

In the sensitivity analyses, the logistic regression analyses was conducted to check the association of Cognitive Impairment using the established FDA thresholds and a ROC-derived Youden cut point as categorical variable in the model.

In the main analysis using established FDA thresholds (Fig. 2B), participants scoring below 60 had 3.35-fold greater odds of Mini-Cog impairment (95% CI, 1.52–7.41) compared with those scoring 75 or higher, and those scoring between 60 and 74 had 1.93-fold increased odds (95% CI, 1.01–3.69). In sensitivity analyses using the ROC-derived Youden cut point of 38, those classified as impaired had 3.95-fold greater odds of Mini-Cog impairment (95% CI, 2.08–7.48) compared with those scoring above the cut point.

## Discussion

This pilot study demonstrates the feasibility and clinical utility of a digitally administered measure, DCTClock, in identifying cognitive impairment in an urban, community-based sample of South Asian adults aged 50 years and older. Notably, despite the absence of subjective memory concerns in nearly all participants (reported by only two individuals), a substantial proportion exhibited impaired performance: 17% based on the Mini-Cog and approximately 42% based on DCTclock scores (<38). This underscores the importance of screening for cognitive impairment for early detection of mild cognitive impairment and dementia. Digital clock drawing performance was strongly associated with a brief paper-and-pencil administered cognitive screening outcome. Participants classified as cognitively impaired by the Mini-Cog scored substantially lower on the DCTclock total score and across all subdomains. In fully adjusted models, each interquartile range higher total DCTclock score was associated with 52% lower odds of impairment.

The strongest associations were observed for spatial reasoning and information processing, in both command and copy conditions. In threshold-based analyses, only the ROC-derived Youden index identified a statistically significant gradient, with individuals scoring below the threshold exhibiting nearly four-fold higher odds of impairment compared to those above it. Age and education were consistently associated with performance across all domains, while sex differences were modest and domain specific. DCTclock was administered in under four minutes, with <1% unanalyzable outputs, and achieved higher completion rates than the Mini-Cog, which showed a 15.5% refusal rate for the clock drawing component. These findings align with prior feasibility studies in high-income settings, where DCTclock acceptance has been > 99% and execution times were minimal, and with large-scale validation efforts linking DCTclock metrics to MRI-derived brain changes and robust MCI discrimination (AUC ~ 0.90)^12,13^.

A key strength of this pilot study is that it represents one of the first evaluations of a tablet-based cognitive assessment in an observational South Asian cohort, addressing a critical gap in the validation of digital tools in low- and middle-income country settings. By pairing DCTclock metrics with an established cognitive screener, the analysis enabled direct benchmarking against standard practice. The analytic approach incorporated interpretable, domain-specific metrics rather than relying solely on composite scores, offering insight into cognitive processes that may signal early impairment^17^. High data completeness, minimal supervision, and low refusal rates support the feasibility of administration in community and primary care contexts.

Several limitations warrant consideration. First, the cross-sectional design limits causal inference and precludes assessment of predictive validity. Second, reliance on the Mini-Cog as the reference standard may have led to under detection of subtle or domain-specific impairments, particularly in participants with limited education. Third, despite adjustment for key demographics, residual confounding from unmeasured factors, such as digital literacy, prior technology exposure, or undiagnosed neurological conditions cannot be excluded. Fourth, the urban sample and small sample size may limit generalizability to rural or lower-access settings. fifth, although completion rates were high, the study did not assess prior tablet use, which may have influenced performance in a small subset of participants. Finally, although most participants had at least some college education, the sample included individuals with lower educational attainment, and this limits generalizability to populations with minimal formal education. In addition, the moderate discriminative performance observed (AUC = 0.669) reflects the expected overlap between two brief screening tools and aligns with the feasibility-focused aims of this pilot rather than diagnostic validation.

In conclusion, this pilot study demonstrates the feasibility and acceptability of a brief tablet-based clock drawing assessment in a South Asian community setting. Although discrimination relative to the Mini-Cog was moderate, DCTclock captured meaningful variation in cognitive performance and produced highly complete data. These findings suggest that digital clock drawing may serve as a scalable adjunct to traditional screening approaches and warrant further evaluation in larger, more diverse, and longitudinal cohorts. As dementia burdens increasingly shift toward low- and middle-income country settings^18^, accessible tools that yield interpretable, high-resolution metrics will be essential for advancing the understanding of early pathophysiological changes, identifying at-risk subgroups, and guiding future longitudinal and biomarker-integrated studies.

## Methods

### CARRS cohort

We conducted a cross-sectional substudy nested within the CARRS (Center for cArdiometabolic Risk Reduction in South Asia) cohort, a large, prospective, population-based study designed to characterize the burden, determinants, and consequences of cardiometabolic diseases in South Asians^19^. Participants aged 20 years and older were recruited from urban households in Delhi and Chennai using a multistage cluster random sampling strategy to obtain a socio-demographically representative sample. Detailed methods have been published previously^20–22^.

CARRS was established in two recruitment waves using identical protocols. CARRS-1 enrolled 12,271 adults between 2010 and 2011, and CARRS-2 enrolled an independent sample of 9591 adults between 2014 and 2016, together comprising more than 21,000 participants. Both cohorts have been followed longitudinally since their inception, with ongoing surveil-lance for clinical and behavioral outcomes. Comprehensive follow-up assessments have been conducted biennially, along with annual tracking of major health events. The latest completed examination cycle was carried out during 2023–2024, representing the seventh follow-up for CARRS-1 and the third for CARRS-2^20–22^. Retention was high, with over 95% of CARRS-1 and 70% of CARRS-2 participants completing at least one follow-up visit^23^.

The CARRS framework uses standardized protocols for data collection, biological sample processing, and geocoded linkage to social and environmental exposures. The study received ethical approval from institutional review boards in India and the United States, and regulatory clearance from the Health Ministry’s Screening Committee, Government of India. All participants provided written informed consent.

We administered DCTClock among a subsample of adults aged 50 years and older who participated in the 2023–2024 follow-up visit in either Delhi (*n* = 150) or Chennai (*n* = 153). Participants were included if they were able to provide consent. Other inclusion criteria required adequate hearing to comprehend the verbal instructions and the to-be-remembered words, and adequate vision and motor use of the dominant hand to be able to see and to draw the stimuli including the clock numbers and hands. Participants who failed to demonstrate any of these abilities during task administration were excluded from further study. Trained field staff administered standardized questionnaires on sociodemographic characteristics and performed the Mini-Cog and the DCT, described below.

### Mini-Cog assessment

During the seventh follow-up of CARRS-1 and the second follow-up of CARRS-2 (2023–2024), the Mini-Cog^6^ screening test was administered to all participants aged 50 years and older. The Mini-Cog is a validated three-minute tool that includes a three-word delayed recall task and a paper-based CDT. It is widely used in clinical practice and in epidemiological studies due to its brevity, lack of copyright and cost requirements, cultural neutrality, and good sensitivity and specificity for detecting cognitive impairment^24,25^.

A recent meta-analysis^26^ of the Mini-Cog observed 76% sensitivity and 83% specificity for detection of dementia, and 84% sensitivity and 79% specificity for MCI. During administration, the individual is first asked to listen carefully to the examiner and repeat out loud three words to remember. They are given three attempts if needed to learn the words. They are then provided with a sheet containing a pre-drawn circle and asked to draw the numbers of a clock and set the hands to “10 past 11.” Instructions can be repeated as needed. After this task, the individual is instructed to say the three words they were asked to remember.

Scores on the Mini-Cog range from 0 to 5. One point is assigned for each spontaneously recalled word (maximum of 3 points). A normal clock is defined as having all numbers placed in the correct sequence and approximate spatial positions (12, 3, 6, and 9 serving as anchors) and clock hands pointing to 11 and 2 to represent 11:10. Hand length is not scored. The Mini-Cog instructions do not mention or recommend double scoring, likely due to the simplicity of scoring. A threshold of ≤2 was used to classify participants as potentially cognitively impaired^6^. This classification was used to describe cognitive status across age, education, and cardiometabolic subgroups.

### DCTclock assessment

Digital cognitive assessment was performed using DCTclock (Linus Health), an FDA-cleared, tablet-based adaptation of the CDT. The assessment was administered on a fourth-generation 11-inch iPad Pro (Apple Inc.) running iOS with Linus Health software version 5.0.5. A fully charged iPad (battery level >50 percent) paired with an Apple Pencil and an updated version of the Linus Tester application was required for administration. The test was conducted in a quiet room free from distractions, with stable internet connectivity to support real-time data synchronization and cloud-based analysis.

Participants completed two tasks within approximately two minutes: the Command condition, in which they were instructed to draw a clock showing “10 past 11,” and the Copy condition, in which they replicated a pre-drawn clock. Time-stamped stylus input captured over 5000 high-resolution features per task. These features were automatically analyzed using an AI-based, cloud-enabled platform, which applies validated algorithms to extract clinically meaningful patterns of behavior. The raw features were integrated into four composite domain scores^9,17^: Drawing Efficiency (overall fluency and economy of pen movement—for instance, the speed and smoothness of strokes relative to drawing size), Information Processing (cognitive response time metrics, including thinking latencies (time between strokes), planning pauses, and task switching speed), Motor Coordination (encompasses both simple and complex motor execution, such as maximum pen velocity and coordination during pen lifts and placements), and Spatial Reasoning (visuoconstructive and spatial layout abilities, such as the accuracy of clock face geometry, numeral positioning, and hand alignment). Consistent with earlier validation work^9^, the primary cognitive outcome in our analysis was the total DCTclock score (range 0–100), calculated from weighted domain composites. Domain-specific scores from both command and copy tasks were analyzed secondarily to assess differential sensitivity across cognitive constructs. These measures were algorithmically derived using the validated Boston Process Approach^27^, integrating time, pressure, and stroke pattern data into interpretable, clinically relevant metrics.

### Statistical analysis

Baseline characteristics were summarized using mean (standard deviation) for normally distributed continuous variables, median (interquartile range) for skewed variables, and counts (percentages) for categorical variables. Comparisons across Mini-Cog impairment groups (score ≤2 vs. ≥3) were conducted using Wilcoxon rank-sum tests for continuous variables and χ^2^ tests for categorical variables.

DCTclock scores and subdomain metrics (drawing efficiency, information processing, motor coordination, and spatial reasoning from both command and copy clocks) were compared based on Mini-Cog cognitive impairment status using non-parametric tests, with accompanying visualizations including density plots and boxplots. The discriminative performance of the overall DCTclock score for identifying cognitive impairment was evaluated using receiver operating characteristic (ROC) curves; area under the curve (AUC) values were calculated with 95% confidence intervals.

To examine the independent associations between DCTclock metrics and cognitive impairment, we fitted generalized linear models with a binomial distribution and logit link function. Each metric was entered as a continuous predictor of binary Mini-Cog impairment (Mini-Cog score <2), in unadjusted models and in models adjusted for age category (50–60, 61–70, >70 years), sex, and education level (≤high school, college, graduate). Results were reported as odds ratios with 95% confidence intervals.

As a sensitivity analysis, the total DCTclock score was categorized using two approaches: (1) U.S. FDA-cleared clinical thresholds (<60, 60–74, ≥75), and (2) a data-derived optimal cut-off based on Youden’s index from the ROC curve. Logistic regression was used to examine associations between these DCTclock categories and Mini-Cog impairment, with findings compared across classification schemes to assess robustness.

All analyses were conducted using R (version 4.3.1). Two-sided P values < 0.05 were considered statistically significant.

## Figures and Tables

**Table 1 |:** Participant Characteristics Stratified by Mini-cog Status

	Overall n = 303	Minicog		P value
		Unimpaired (n = 252)	Impaired (n = 51)	
Current Age, Years	62.3 (8.94)	61.8 (8.74)	65.0 (9.55)	0.019
Age categories, n (%)				0.070
- 50 to 60	144 (47.5)	125 (49.6)	19 (37.3)	
- 61–70	103 (34.0)	86 (34.1)	17 (33.3)	
- >70	56 (18.5)	41 (16.3)	15 (29.4)	
Sex, Female, n (%)	168 (55.4)	135 (53.6)	33 (64.7)	0.192
Education				0.148
- Up to high school	23 (7.6)	17 (6.7)	6 (11.8)	
- College	197 (65.0)	161 (63.9)	36 (70.6)	
- Graduate	83 (27.4)	74 (29.4)	9 (17.6)	
City				0.318
- Chennai	153 (50.5)	131 (52.0)	22 (43.1)	
- Delhi	150 (49.5)	121 (48.0)	29 (56.9)	
Current history of memory issues, n (%)^a^	1 (0.3)	NA	1 (2)	NA
Memory medication, n (%)^b^	1 (0.3)	NA	1 (2)	NA

a Memory impairment was self-reported as a prior diagnosis of mild cognitive impairment or dementia.

b Memory medication refers to prescription cognitive enhancers, such as anticholinesterase inhibitors (for example, donepezil/Aricept).

**Table 2 |:** Associations of Age, Sex, and Education with DCTclock Total Score and Subdomain Scores in the Command and Copy Tasks

Outcome	Median (IQR)	Estimate (SE)	P value
DCTClock Score			
Age (years)			
- 50 to 60	54.6 (33.5, 74.1)	Ref	
- 61–70	43.7 (18.6, 63.5)	−10.91 (3.26)	<0.001
->70	24.5 (12.1, 50.5)	−21.27 (3.97)	<0.001
Sex			
- Male	47.2 (22.6, 64.2)	Ref	
- Female	46.1 (18.6, 68.3)	−0.24 (3.06)	0.938
Education			
- Up to high school	19 (7.7, 36.9)	Ref	
- College	44.2 (18.7, 60.3)	17.86 (5.68)	0.002
- Graduate	59.2 (34.9, 76.1)	30.07 (6.06)	<0.001
Drawing efficiency (Command domain)			
Age (years)			
- 50 to 60	56.6 (46.9, 63.8)	Ref	
- 61–70	54.3 (43.8, 62.9)	−2.50 (1.76)	0.158
- >70	51.6 (40.1, 59.4)	−5.16 (2.15)	0.017
Sex			
- Male	56.5 (46.8, 63.5)	Ref	
- Female	54 (43.5, 62.2)	−2.44 (1.59)	0.125
Education			
- Up to high school	42.3 (35.3, 53.1)	Ref	
- College	54.6 (43.3, 61.4)	8.14 (2.96)	0.006
- Graduate	59.3 (52.9, 65.7)	14.91 (3.16)	<0.001
Information Processing (Command domain)			
Age (years)			
- 50 to 60	54.3 (47.8, 61.2)	Ref	
- 61–70	53.1 (46.8, 61.6)	−1.32 (1.50)	0.38
- >70	53.6 (46.1, 60.5)	0.13 (1.83)	0.945
Sex			
- Male	54.7 (49.8, 61.3)	Ref	
- Female	52.6 (44.6, 61)	−2.98 (1.34)	0.026
Education			
- Up to high school	38.8 (29.6, 46.9)	Ref	
- College	53.2 (46.6, 59.9)	12.29 (2.38)	<0.001
- Graduate	60.8 (52.9, 66.1)	19.13 (2.54)	<0.001
Simple Motor (Command domain)			
Age (years)			
- 50 to 60	56.6 (50.9, 63.2)	Ref	
- 61–70	56.4 (48.7, 62.5)	−0.82 (1.31)	0.532
- >70	51.2 (40.7, 60.5)	−6.20 (1.60)	<0.001
Sex			
- Male	56.2 (48.3, 62.6)	Ref	
- Female	56.1 (49.1, 62.7)	0.94 (1.20)	0.433
Education			
- Up to high school	55.5 (48.5, 62.4)	Ref	
- College	56.1 (49.4, 62.6)	−1.12 (2.34)	0.633
- Graduate	56.2 (48.7, 62.9)	−1.10 (2.49)	0.659
Spatial Reasoning (Command domain)			
Age (years)			
- 50 to 60	58.4 (36.8, 69.8)	Ref	
- 61–70	50.0 (31.9, 66.9)	−5.00 (2.76)	0.071
->70	39.5 (20.7, 55.5)	−13.20 (3.36)	<0.001
Sex			
- Male	51.9 (33.1, 63.9)	Ref	
- Female	52.3 (31.7, 68.7)	0.79 (2.53)	0.754
Education			
- Up to high school	24.3 (17.2, 41.9)	Ref	
- College	50 (31.5, 64.5)	15.03 (4.69)	0.002
- Graduate	61.3 (41.4, 74.4)	25.28 (5.00)	<0.001
Drawing efficiency (Copy domain)			
Age (years)			
- 50 to 60	57.4 (52.4, 61.7)	Ref	
- 61–70	53.1 (45.2, 59.3)	−4.48 (1.46)	0.002
- >70	47 (38.7, 57.4)	−9.12 (1.78)	<0.001
Sex			
- Male	56.2 (47.3, 61.6)	Ref	
- Female	54.9 (46, 59.9)	−2.25 (1.36)	0.099
Education			
- Up to high school	48.5 (29.2, 54.4)	Ref	
- College	55.9 (45, 60.4)	8.14 (2.53)	0.001
- Graduate	56.5 (50.2, 62.3)	11.31 (2.70)	<0.001
Information Processing (Copy domain)			
Age (years)			
- 50 to 60	51.2 (41, 59)	Ref	
- 61–70	49.5 (39.9, 58.8)	−0.69 (1.76)	0.695
- >70	48.3 (40.2, 55)	−2.72 (2.15)	0.207
Sex			
- Male	51.6 (42.8, 60.1)	Ref	
- Female	48.6 (39.1, 57.3)	−3.47 (1.57)	0.028
Education			
- Up to high school	35.3 (20.4, 43.6)	Ref	
- College	49 (40.5, 57)	13.29 (2.82)	<0.001
- Graduate	54.5 (47.3, 63.1)	19.43 (3.01)	<0.001
Simple Motor (Copy domain)			
Age (years)			
- 50 to 60	55.4 (50.6, 61.3)	Ref	
- 61–70	54.6 (49.5, 58.8)	−1.72 (0.97)	0.076
- >70	49.4 (44.4, 53.3)	−6.14 (1.18)	<0.001
Sex			
- Male	54 (47.7, 59.7)	Ref	
- Female	54.4 (49.7, 58.8)	0.75 (0.90)	0.407
Education			
- Up to high school	54.6 (48.7, 57.2)	Ref	
- College	54.3 (48.5, 60.2)	0.22 (1.72)	0.899
- Graduate	54.4 (49.5, 59)	−0.13 (1.84)	0.942
Spatial Reasoning (Copy domain)			
Age (years)			
- 50 to 60	61.2 (44.8, 73.2)	Ref	
- 61–70	45.4 (27.9, 63.3)	−12.83 (2.78)	<0.001
- >70	34.4 (24.1, 60.7)	−16.63 (3.39)	<0.001
Sex			
- Male	51.2 (31.4, 68.7)	Ref	
- Female	50.8 (31.8, 69.8)	1.40 (2.62)	0.594
Education			
- Up to high school	35.2 (19.8, 55)	Ref	
- College	49.5 (31.3, 68.5)	10.91 (4.92)	0.027
- Graduate	59.4 (38.4, 73.3)	16.15 (5.26)	0.002

Median (interquartile range, IQR) values are shown for each subgroup. Linear regression models were used to estimate the associations of age (reference: 50 to 60 years), sex (reference: male), and education (reference: up to high school) with each DCTclock metric. The Estimate represents the difference in score compared to the reference group, along with standard errors (SE) and p-values. Results are presented for the DCTclock total score and for each subdomain: Drawing efficiency, information processing, simple motor, and spatial reasoning, separately for the command and copy conditions.

A positive estimate indicates better performance relative to the reference group. Statistically significant differences (*p* < 0.05) are bolded in the table.

## Data Availability

The datasets generated and/or analyzed during the current study are part of the *Center for cArdiometabolic Risk Reduction in South Asia (CARRS)* cohort and are not publicly available due to institutional and ethical restrictions on data sharing. De-identified data may be made available to qualified researchers upon reasonable request to the corresponding author (JR; [ram.jagannathan@emory.edu](mailto:ram.jagannathan@emory.edu)) and subject to approval by the CARRS data governance committee.
